# Extracellular vesicles and particles as mediators of long-range communication in cancer: connecting biological function to clinical applications

**DOI:** 10.20517/evcna.2023.37

**Published:** 2023-08-17

**Authors:** Tetsuhiko Asao, Gabriel Cardial Tobias, Serena Lucotti, David R. Jones, Irina Matei, David Lyden

**Affiliations:** ^1^Children’s Cancer and Blood Foundation Laboratories, Departments of Pediatrics, Cell & Developmental Biology, Drukier Institute for Children’s Health and Meyer Cancer Center, Weill Cornell Medicine, New York, NY 10021, USA.; ^2^Thoracic Service, Department of Surgery, Memorial Sloan Kettering Cancer Center, New York, NY 10065, USA.; ^3^Department of Respiratory Medicine, Juntendo University Graduate School of Medicine, Tokyo 163-8001, Japan.; ^#^These authors contributed equally.

**Keywords:** Extracellular vesicles and particles, cancer, pre-metastatic niche, organotropism, biomarkers, treatment

## Abstract

Over the past decade, extracellular vesicles and particles (EVPs) have emerged as critical mediators of intercellular communication, participating in numerous physiological and pathological processes. In the context of cancer, EVPs exert local effects, such as increased invasiveness, motility, and reprogramming of tumor stroma, as well as systemic effects, including pre-metastatic niche formation, determining organotropism, promoting metastasis and altering the homeostasis of various organs and systems, such as the liver, muscle, and circulatory system. This review provides an overview of the critical advances in EVP research during the past decade, highlighting the heterogeneity of EVPs, their roles in intercellular communication, cancer progression, and metastasis. Moreover, the clinical potential of systemic EVPs as useful cancer biomarkers and therapeutic agents is explored. Last but not least, the progress in EVP analysis technologies that have facilitated these discoveries is discussed, which may further propel EVP research in the future.

## INTRODUCTION

EVPs are nano-sized structures secreted by all cell types that serve as critical mediators of intercellular communication^[1]^. They participate in a wide array of physiological processes, such as immune responses, neural signaling, and tissue regeneration, as well as pathologies, including cancer, inflammation, infectious diseases, neurological disorders, cardiovascular diseases, and autoimmune disorders. The groundbreaking research into the mechanisms that govern vesicle trafficking, which is crucial for understanding EVP biogenesis, earned the 2013 Nobel Prize in Physiology or Medicine^[2]^.

**Figure 1:**
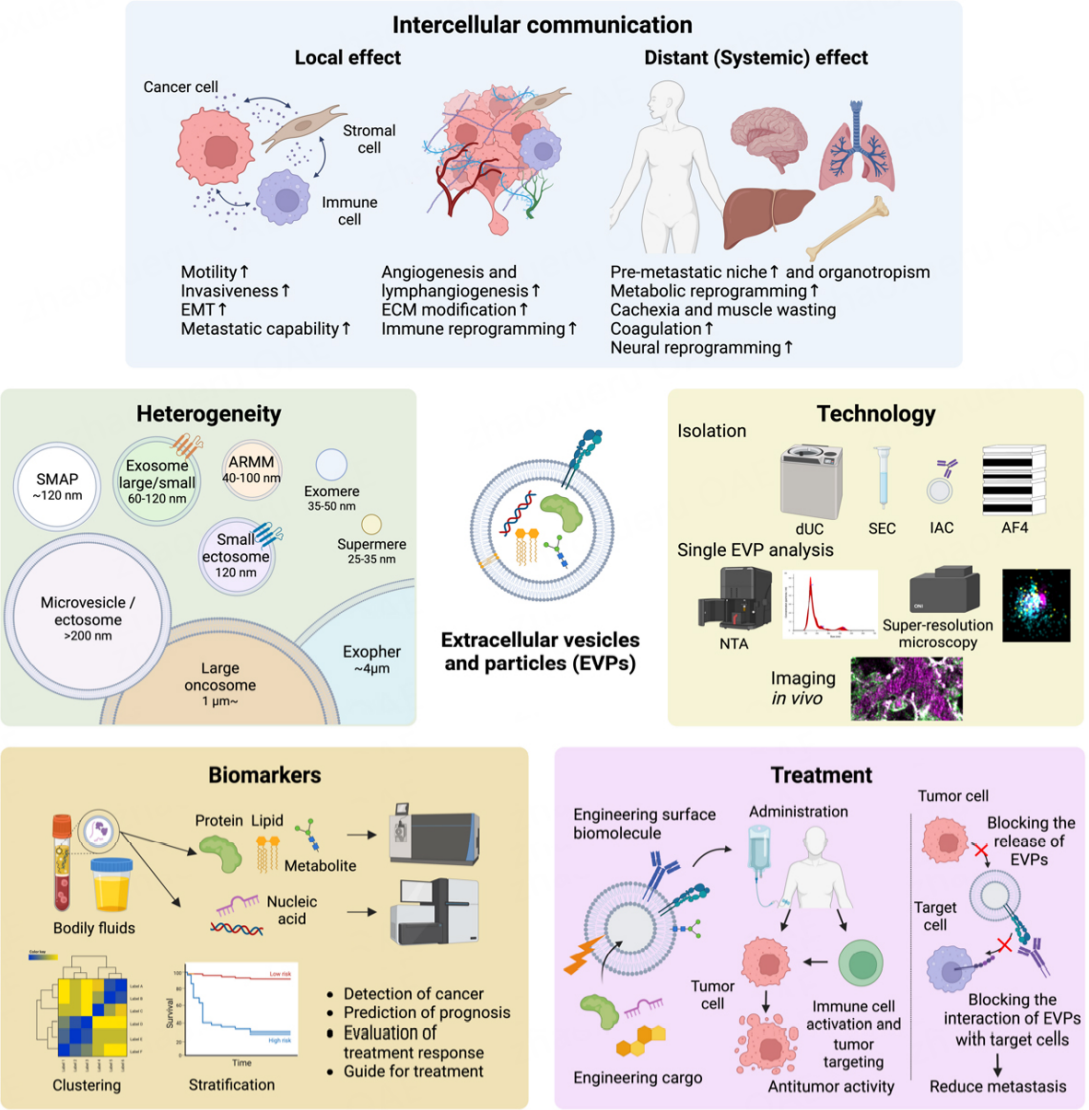
Overview of extracellular vesicles and particle (EVP)-focused fields of study in cancer research. *Intercellular communication*: EVPs mediate intercellular communication between cancer, stromal, and immune cells by transferring various biomolecules to nearby as well as distant organs, resulting in both local and systemic effects. Cancer-derived EVPs stimulate angiogenesis, promote tumor growth, and suppress antitumor immunity, enhancing tumor invasion and metastatic potential. *Heterogeneity*: EVPs are highly diverse and new populations are constantly being discovered. The classification of new EVP populations requires multifaceted evaluation, including size, composition, membrane surface markers, and origin. *Technology*: various methods for separating EVPs, including differential ultracentrifugation, size exclusion chromatography, immunoaffinity capture, and asymmetric flow field flow fractionation, are available and they have their own advantages and disadvantages. Furthermore, analyses at the single EVP level, such as nanoparticle tracking analysis, high-sensitivity flow cytometry, high-resolution microscopy, as well as *in vivo* imaging, are now introduced. *Biomarkers*: EVPs in bodily fluids show potential as non-invasive biomarkers for early diagnosis, treatment response, and prognosis of cancer. Their constituent proteins, nucleic acids, lipids, and metabolites can be isolated and analyzed by mass spectrometry and next-generation sequencing. *Treatment*: EVPs can be used therapeutically by administering EVPs isolated from specific cells, loading various biomolecules such as anticancer or molecularly targeted drugs, or by engineering EVPs to enhance function. Strategies that block the production of tumor-derived EVPs or block the interaction of EVPs with target cells are also being investigated. EMT: epithelial-mesenchymal transition; ECM: extracellular matrix; SMAP: supramolecular attack particles; ARMM: arrestin domain-containing protein 1 -mediated microvesicles; dUC: differential ultracentrifugation; SEC: size exclusion chromatography; IAC: immunoaffinity capture; AF4: asymmetric flow field flow fractionation; NTA: nanoparticle tracking analysis.

Over the past decade, EVP research has made remarkable leaps, which can be attributed to the solid foundation laid by these studies [Figure 1]. Advances have been achieved in several areas, encompassing the discovery of new EVP fractions facilitated by technological innovations, comprehensive characterization of EVP cargo, such as proteins, nucleic acids, and metabolites, and a better grasp of their involvement in various diseases. Particularly noteworthy is elucidating the roles of EVPs in cancer development, such as in pre-metastatic niche (PMN) formation, the creation of a favorable environment within distant organs that supports cancer cell colonization, as well as metastatic organotropism and progression. More recently, EVPs have been shown to play critical roles in mediating the systemic effects of cancer, leading to vascular dysfunction, coagulation, metabolic alterations, and immune suppression. These findings have greatly enhanced our knowledge in the field and led to the recognition of EVP cargo as biomarkers of cancer, its progression and response to treatment, as well as the exploration of the therapeutic applications of EVPs.

This review aims to provide a comprehensive overview of the research conducted on the pleiotropic roles of EVPs in cancer over the past decade, highlighting the most significant discoveries and their implications for the future of cancer diagnosis and treatment.

## EVP HETEROGENEITY

EVPs are highly heterogeneous and include a wide variety of particles, ranging from about 10 nm to over 1 μm in diameter. Since their discovery in the 1980s, research on extracellular vesicles has revealed that all cell types release a variety of EVPs. Conventionally, extracellular vesicles have been largely categorized as exosomes and microvesicles (MVs) based on size and biogenesis pathways^[3]^.

EVPs carry various cargo, such as proteins, nucleic acids (RNA, DNA), lipids, and metabolites, and can exert various functions. Recent advances in technology have led to the discovery of novel EVP populations, as well as the reclassification of conventional EVPs^[4-13]^. In particular, small EVPs were recently re-classified based on their size and structure as large exosomes (Exo-L; 90-120 nm), small exosome (Exo-S; 60-90 nm), non-membranous nanoparticles termed exomeres (35-50 nm) and supermeres (25-35 nm)^[12-14]^. Exomeres and supermeres are nanoparticles that lack an external membrane structure and are therefore classified as new populations called extracellular nanoparticles (ENPs). Each of these EVP subsets has a distinct protein, nucleic acid, lipid, and proteoglycan composition, suggesting that they have distinct functions. Exomeres are rich in metabolic enzymes, particularly those involved in glycolysis, and may play a role in metabolic programs, coagulation, and responses to low oxygen. Moreover, cancer-derived supermeres promote lactate secretion and drug resistance, and decrease hepatic lipids and glycogen, suggesting that ENPs may be involved in the regulation of systemic metabolism. More recently, another small extracellular vesicle subpopulation has been described, the small ectosomes, approximately 120 nm size particles that bud from the plasma membrane^[8]^. In contrast to exosomes, which express CD63 but little CD9, ectosomes express CD9 and CD81 but little CD63. This finding not only challenges the conventional assumption that tetraspanins (CD9, CD63, CD81) serve as ubiquitous surface markers for exosomes, but also highlights the need to redefine our understanding of ectosomes as a small EVP subset.

The complexity of EVP subpopulations and classification is constantly evolving, as many other subsets have been defined based on size, function and cargo, or the cell type/organ or even species they derive from. Arrestin domain-containing protein 1 (ARRDC1)-mediated MVs (ARMMs) are small vesicles (40-100 nm) that bud directly from the plasma membrane in an ARRDC1- and tumor susceptibility 101 (TSG101)-dependent manner and which are involved in signaling through the non-canonical intercellular Notch signaling pathway^[4,15]^. In turn, supramolecular attack particles (SMAPs) are immune system-specific particles of approximately 120 nm secreted by cytotoxic T lymphocytes, which deliver proteins, such as perforins and granzymes, to target cells and have autonomous cytotoxicity^[5]^. Mitovesicles are small EVPs isolated from brain tissue that are rich in mitochondrial proteins, lipids, and DNA, and have been implicated in mitochondrial abnormalities associated with Down syndrome^[6]^. Large oncosomes are vesicles 1-10 µm in size shed by tumor cells that promote tumor progression^[9,10]^. Exophers are 4 µm-sized vesicles secreted from nematode (*C. elegans*) neurons containing proteins and organelles that are secreted as a type of cellular stress response to maintain cellular proteostasis^[11]^. Taken together, these findings demonstrate that EVPs exhibit diversity not only in size, but also in their origin, targets, and functions. Since some of these categories overlap or are not fully defined, and in response to the increasing number of EVP subsets, the International Society for Extracellular Vesicles (ISEV) has established the Minimal Information for Studies of Extracellular Vesicles (MISEV) guidelines to provide a standardized set of reporting criteria for characterizing EVPs^[16]^. The classification of EVPs is still evolving, and international consensus is being discussed and updated.

Although much remains unknown regarding the biogenesis, target cells and organs, as well as the function of newly identified EVPs, ongoing research suggests that these properties may also be unique to each EVP subset. Additionally, EVPs may possess yet-to-be-defined physiological and pathological functions that transcend the limits of conventional classification, highlighting their highly diverse nature and central roles in intercellular communication throughout the animal kingdom and evolution.

## EVP ROLES IN INTERCELLULAR COMMUNICATION

As an intercellular communication system, EVPs may be the most complex, as they can horizontally transfer a wide range of biomolecules to target cells. As a result, EVPs are essential means of information transfer in a wide range of physiological processes, such as immunity, neurology, tissue regeneration, and development, as well as pathological processes, such as cancer, inflammation, infection, neurological diseases, cardiovascular and metabolic diseases, and autoimmune diseases^[17-19]^. Thus, while EVPs are involved in communication between various cells during normal development and physiology, their best-characterized functions are in cancer. As such, EVPs can transfer information between cancer cells and immune cells, epithelial cells, stromal cells, neurons, and even pathogens^[17-19]^. Pioneering studies in the late 2000s demonstrated that intercellular communication occurs via the transfer of EVP content^[20-22]^. For example, del Conde *et al*. showed that tissue factors in EVPs derived from monocytes or macrophages are transferred to platelets and activate the coagulation process, while Ratajczak *et al*. showed that EVPs derived from embryonic stem cells are enriched in transcription factor mRNA, which can be delivered to target cells and translated into the corresponding proteins^[20,21]^. The biomolecules delivered by EVPs, including proteins, RNA (mRNA, miRNA, long non-coding RNA), DNA, lipids, and metabolites, can have various effects on target cells and can modify their characteristics^[23-25]^. This horizontal transfer of information through EVPs occurs not only locally but also systemically, as EVPs can deliver biomolecules to distant organs. For example, EVPs derived from melanoma can transfer the Met oncoprotein to progenitor cells in the bone marrow, reprogramming them and leading to their egress from the bone marrow and migration to future sites of metastasis where they contribute to pre-metastatic niche formation^[25]^. Moreover, biomolecules delivered by EVPs exert various effects on target cells, ranging from direct and short-term effects through the transfer of molecules, such as active proteins involved in signal transduction and non-coding RNAs involved in transcriptional regulation, to long-term effects through target cell reprogramming. As such, bone marrow cells that have been educated and reprogrammed by tumor-derived EVPs can maintain their altered function upon serial transplantation (whereby donor hematopoietic stem cells are engrafted into a primary host and subsequently isolated and engrafted into secondary hosts) in mice, suggesting the long-term impact of EVPs-mediated reprogramming is crucial for pathological process^[25]^. EVP cargo is only to a certain extent reflective of the cell of origin, and many molecules are enriched and thus selectively packaged. For instance, it is known that integrin molecules are differentially packaged into EVPs derived from various cancers^[26]^. Although the exact mechanism leading to selective packaging remains still unknown, recent studies have shed light on how nucleic acids, such as miRNA and DNA, are loaded into EVPs^[27,28]^.

In summary, EVPs play a crucial role in intercellular communication and have emerged as an indispensable means of information transfer, both physiologically and pathologically, alongside soluble factors.

## METASTASIS: LOCAL AND DISTANT EFFECTS OF EVPs

### Local effects of EVPs

EVPs play a significant role in both local and systemic effects of cancer progression and metastasis^[29,30]^. Locally, they mediate intercellular communication within the tumor microenvironment, transferring oncogenic proteins^[25,31,32]^, lipids, and nucleic acids to recipient cells, such as cancer cells^[33]^, cancer-associated fibroblasts (CAFs), macrophages^[34,35]^, and endothelial cells^[36]^. A key discovery linking EVPs to metastasis emerged in 2012 when researchers found that CAF-derived EVPs could promote metastasis by transferring their contents to cancer cells^[37]^, establishing a new paradigm in the field and opening the door to further investigations into the specific mechanisms through which EVPs contribute to cancer progression. In particular, the CD81 tetraspanin, present in CAF-derived EVPs, is a key factor in inducing breast cancer cell motility and promoting metastasis *in vivo*^[37]^, through activation of the planar cell polarity (PCP) pathway, which is intimately connected with the Wnt signaling pathway^[38]^. Wnt signaling is a highly conserved cellular signaling system that plays essential roles in regulating cell proliferation, differentiation, and migration, as well as PCP^[39,40]^. The interplay between Wnt signaling and PCP regulation also influences the formation and function of invadopodia^[41-43]^, finger-like protrusions that facilitate tumor cell invasion^[41-44]^. These invadopodia have been shown to be crucial for exosome secretion^[45]^, further emphasizing the importance of Wnt signaling and PCP regulation in the context of cancer metastasis. Additionally, several other tetraspanins present in EVPs also contribute to metastasis through various signaling pathways^[46]^, such as the integrin, epidermal growth factor receptor (EGFR), focal adhesion kinase (FAK), phosphoinositide 3-kinase (PI3K)/protein kinase B (Akt), and mitogen-activated protein kinase (MAPK) pathways^[46-48]^. Numerous other landmark studies revealed the functions of other EVP-associated proteins and their potential roles in cancer progression^[25,26,37,49-51]^.

Subsequent research focused on how EVPs contribute to metastasis through various mechanisms, such as promoting angiogenesis^[25,52]^, modulating immune responses^[53-56]^, and inducing epithelial-to-mesenchymal transition (EMT)^[57-59]^. Moreover, EVPs could transfer oncogenic proteins and nucleic acids, including microRNAs and long non-coding RNAs, to recipient cells, modulating their gene expression and influencing their proliferation, migration, and invasion capabilities, ultimately contributing to metastatic potential^[33,52,60-63]^. This process, known as EVP-mediated horizontal transfer, enabled cancer cells to communicate with and alter the behavior of neighboring cells in the tumor microenvironment, contributing to the metastatic process.

Building on these foundational findings, additional landmark studies have continued to reveal the local effects of EVPs on cancer-promoting processes, such as extracellular matrix (ECM) remodeling, inflammation, and rewiring of oncogenic pathways, further elucidating the multifaceted roles of EVPs in cancer progression and metastasis. For example, a study by Nabet *et al.* demonstrated that dynamic feedback between tumor and stroma subverts normal inflammatory responses by triggering the release of EVPs containing unshielded RNAs that activate pattern recognition receptors, thereby promoting tumor growth and metastasis^[64]^. Inflammatory pathways, normally activated in response to viral RNAs, were induced in cancer by deregulated release of exposed endogenous 5’-triphosphorylated double-stranded RNA in tumor stroma EVPs^[64]^. These unshielded non-coding RNAs enriched in EVPs from tumor-activated stromal cells acted as damage-associated molecular patterns (DAMPs) signals, triggering a feedback loop that activated the pattern recognition receptor retinoic acid-inducible gene I (RIG-I) and interferon-stimulated genes in breast cancer and innate immune cells^[64]^. In light of these findings, further investigation is warranted to elucidate the complex interplay between tumor-elicited systemic interferon responses induced by EVP DAMP proteins, and nucleic acids (DNA and RNA), as well as to determine the context-dependent roles of these responses in promoting or inhibiting tumorigenesis and metastasis, potentially considering factors such as the timing and duration of interferon activation both locally and systemically.

Additionally, recent studies have focused on the impact of ECM stiffness on EVP secretion and cargo, as ECM stiffening can trigger tumor growth, invasiveness, and chemoresistance, leading to a direct correlation between tumor aggressiveness and metastatic potential^[65]^. Wu *et al.* have shed light on the mechanisms linking ECM stiffness with EVP secretion, demonstrating that ECM stiffness activates FAK/PI3K/Akt signaling, promoting Rab8-induced small extracellular vesicle secretion, and thus cancer growth^[65]^. Moreover, stiff ECM enhanced the secretion of EVPs and altered their cargo, specifically in the context of hepatocellular carcinoma (HCC), which is strongly associated with increased collagen deposition and tissue stiffness^[65]^.

Finally, EVPs can also rewire oncogenic signaling locally within the primary tumor as well as metastasis. Astrocyte-derived EVPs containing microRNA-19a induce the loss of phosphatase and tensin homolog (PTEN) expression in tumor cells, promoting metastasis^[63]^. PTEN loss in brain metastases was found to be reversible and induced by the brain microenvironment, rather than originating from PTEN-low cells in the primary tumor. The pathway linking PTEN loss to the promotion of brain metastasis involved nuclear factor-κB signaling and C-C motif chemokine 2 (CCL2)-induced myeloid cell recruitment, indicating reciprocal crosstalk between tumor cells and the brain microenvironment. Finally, in the case of head and neck cancers with mutant p53, EVPs secreted by cancer cells lack a specific miRNA called miR-34a, which is a tumor suppressor. The absence of miR-34a in these EVPs drives the formation of new neurons in the tumor microenvironment, contributing to tumor growth and decreased survival time^[66]^. Specifically, it enables sympathetic neurons to fuel tumor proliferation via noradrenaline release while allowing parasympathetic neurons to spur cancer cell invasion and migration via acetylcholine^[67]^. This groundbreaking discovery has opened up new avenues of research to better understand the mechanisms through which EVPs can influence the formation of brain metastases and identify potential therapeutic targets to prevent or treat such metastases.

### Distant effects of EVPs

**Figure 2:**
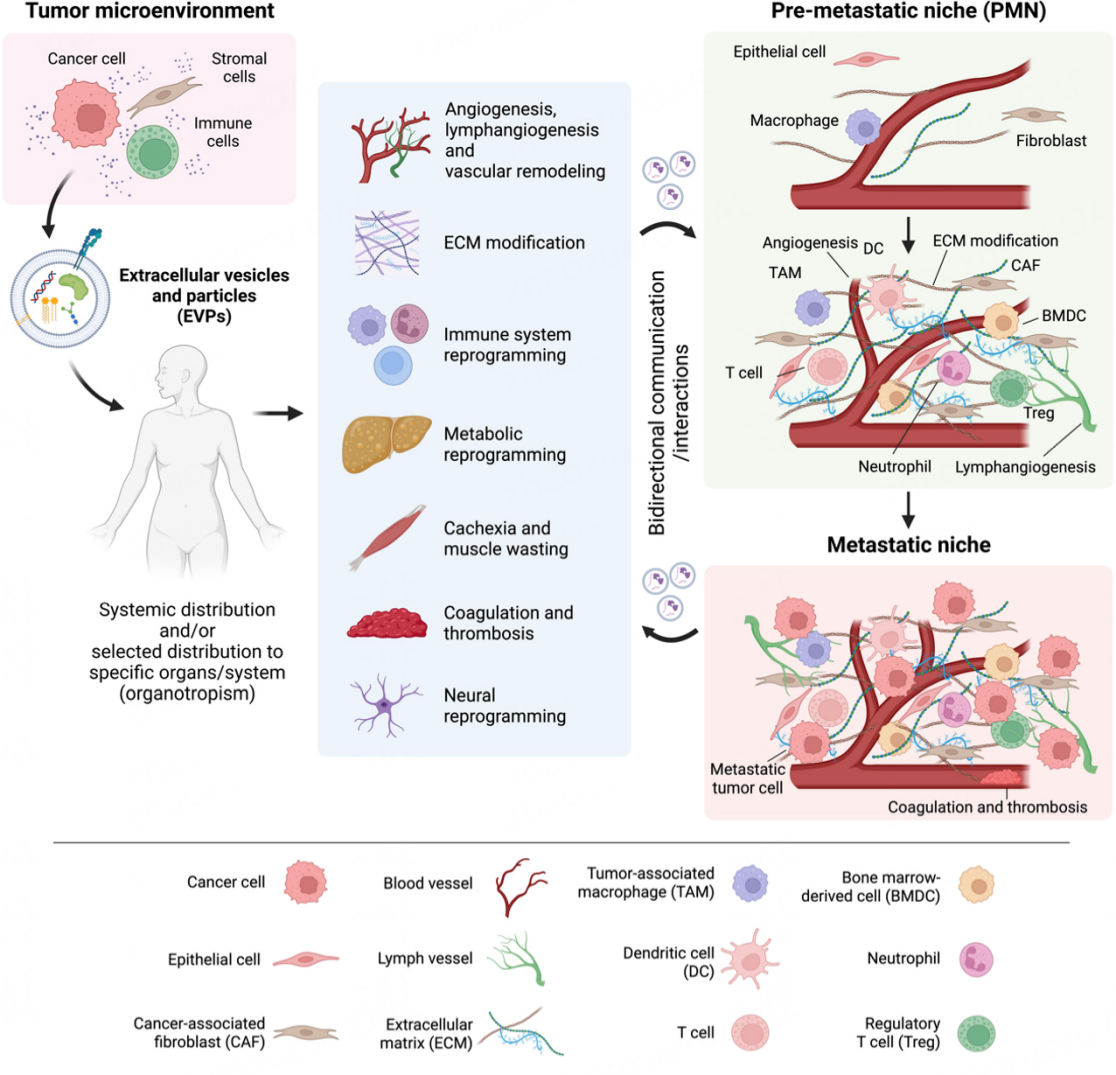
The systemic effects of extracellular vesicles and particles (EVPs) in cancer. EVPs can transport various biomolecules to distant organs. EVP uptake by specific cells in the microenvironment of the remote organ results in functional reprograming of target cells. For example, tumor-derived EVPs can promote angiogenesis and vascular remodeling, modify the extracellular matrix (ECM), promote lymphangiogenesis, modulate the function of immune cells to create an immunosuppressive environment, and reprogram metabolism. Moreover, EVPs induce cachexia, muscle wasting, coagulation and thrombosis, and neural reprogramming. These alterations contribute to the formation of a pre-metastatic niche (PMN) that facilitates cancer colonization and metastasis by recruiting bone marrow-derived cells (BMDCs) to modify the microenvironment, immune cells that suppress tumor immunity such as tumor-associated macrophages (TAMs) and regulatory T cells (Tregs), and neutrophils that promote metastasis. Dysregulation can also occur in T cells, dendritic cells (DC), and natural killer cells. Thus, the PMN becomes an immunosuppressed, metastasis-supporting environment. Once metastasis has taken place, the PMN transitions to a metastatic niche where angiogenesis, ECM remodeling, and progression of an immunosuppressive environment continue. In addition to directly modifying the microenvironment, tumor cells, stromal cells, and immune cells release EVPs that facilitate bidirectional communication/interaction. CAF: cancer-associated fibroblast.

As EVPs readily circulate via all bodily fluids, they have a widespread and long-range impact on various organ systems. Tumor-derived EVPs play a critical role in establishing PMN in distant organs, as they modify the behavior of recipient cells and alter vascular function, coagulation, metabolism, immunity, and the nervous system, thereby promoting cancer cell colonization and metastasis [Figure 2]. Notably, EVPs can traverse and permeabilize the blood-brain barrier, allowing cancer cells to infiltrate the brain-an area typically regarded as immune-privileged.

#### Pre-metastatic niche

EVPs play a crucial role in the formation of the pre-metastatic niche (PMN), which is an altered microenvironment supportive of tumor cell engraftment at distal sites of future metastasis. PMN formation is a multi-step process, which includes angiogenesis, vascular leakiness, ECM and stromal remodeling, lymphangiogenesis, and changes in the immune microenvironment^[68]^. These processes create an environment suitable for cancer cell colonization^[49,69-71]^. It has been shown in various mouse models of lung, brain, and liver metastasis that proteins and nucleic acids contained in EVPs reach distant organs and promote PMN formation^[25,72-75]^. In a 2012 study, Peinado *et al*. demonstrated that melanoma-derived EVPs indirectly facilitate the formation of the PMN by recruiting proangiogenic bone marrow-derived cells to future sites of metastasis^[25]^. Melanoma-derived EVPs, packaging Met oncoprotein, can travel to the bone marrow, where they are taken up by bone marrow-derived cells (BMDCs), promoting their migration to the lungs, where they facilitate lung metastasis by inducing angiogenesis, vascular leakage, and ECM remodeling^[25]^. This research emphasized the importance of EVP-mediated long-range communication in promoting metastasis^[25]^. These findings were reinforced by studies on pancreatic cancer-derived EVPs, which can directly promote liver PMN formation organs^[72]^. Moreover, pancreatic ductal adenocarcinoma is a CAF-rich tumor, and CAF-derived EVPs play a significant role in promoting the formation of PMN at distant metastatic sites such as the lung through the release of EVPs^[50]^. Thus, EVPs orchestrate PMN formation in a cancer-type specific manner, targeting those organs that are the most common sites of metastasis through direct and indirect mechanisms.

The role of EVPs in breaching the blood-brain barrier (BBB) has also gained attention in the context of metastasis. In 2019, Rodrigues *et al.* demonstrated that breast cancer-derived EVPs could cross the BBB and promote brain metastasis^[75]^. Mechanistically, breast cancer-derived EVPs carrying cell migration-inducing and hyaluronan-binding protein (CEMIP) reach the brain, where they are taken up by endothelial cells and microglia, remodeling the peri-vascular niche and inducing neuroinflammation, both processes that support tumor cell survival in the hostile brain environment and ultimately lead to brain metastasis^[75]^. Similarly, cancer-derived EVPs containing small nuclear RNAs are taken up by alveolar epithelial cells in the lung, which upregulate Toll-like receptor expression, enhancing neutrophil recruitment and subsequently promoting lung metastasis^[74]^. EVPs also play critical roles in lymph node metastasis, as malignant melanoma-derived EVPs containing nerve growth factor receptors spread in the lymphatic system and are taken up by lymphatic endothelial cells, promoting lymphangiogenesis and tumor colonization^[73]^.

In summary, the past decade has brought a clear understanding that cancer-related EVPs deliver various biomolecules to target cells, adjusting the microenvironment of the future organ of metastasis before metastasis occurs, and promoting cancer cell engraftment, revolutionizing our understanding of cancer metastasis by shifting the focus away from cell-intrinsic mechanisms of metastasis and towards systemic interactions with the microenvironment of various organs.

#### Organotropism

**Figure 3:**
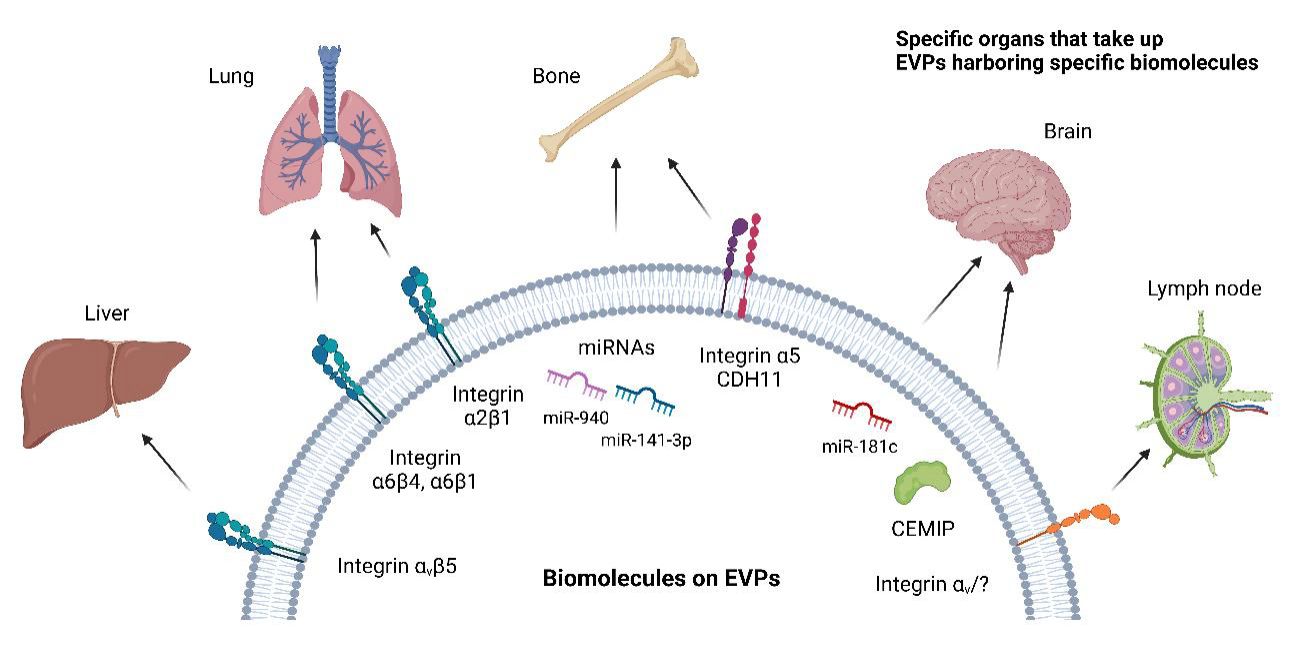
Extracellular vesicles and particles (EVPs) determine metastatic organotropism. Specific molecules expressed on EVPs interact with cells or the extracellular matrix of specific organs to deliver biomolecules to distant organs. This causes changes in the microenvironment, leading to pre-metastatic niche (PMN) formation and facilitating distant metastasis to specific organs. For example, tumor-derived EVPs expressing integrin αvβ5 are taken up by the liver and promote liver PMN formation. Similarly, integrin α6β1 and α6β4 expressed on tumor-derived EVPs are taken up by the lung and promote lung metastasis. Integrin α2β1 on cancer-associated fibroblast-derived EVPs also promotes lung metastasis. Integrin α5, miR-940, and miR-141-3p are involved in bone metastasis, CEMIP and miR-181c in brain metastasis, and integrin αv in lymph node metastasis, as reported. CDH11: cadherin 11; CEMIP: cell migration inducing hyaluronidase 1.

The concept of metastatic organotropism, which can be traced back to the late 19th century, was first proposed by Stephen Paget in his 1889 “seed and soil” hypothesis. Paget attempted to explain the non-random pattern of metastatic cancer dissemination by suggesting that the tumor cells, or “seeds,” have a preferential affinity for specific organs, or “soil”^[76]^. This hypothesis laid the groundwork for understanding the complex and highly regulated process of metastasis, which is now a critical factor in cancer patient staging and prognosis^[77]^. Importantly, while tumor-derived EVPs can be detected in various bodily fluids and interact with a wide range of cell types, they are not uniformly taken up in all organs^[12,26,78]^. The selective uptake of tumor-derived EVPs by specific organs results from a combination of factors, such as EVP cargo, the composition of the tumor-derived EVP membrane, the recipient cell’s surface receptors, and the presence of specific proteins and ligands on both the vesicle and target cell surfaces^[12,26,78]^. In addition, the physiological state and microenvironment of the recipient organ can impact the ability of tumor-derived EVPs to interact with and be internalized by cells, resulting in a heterogeneous distribution and uptake across different organs. Thus, the selective uptake of tumor-derived EVPs aligns with Paget’s “seed and soil” hypothesis, as it emphasizes the critical role of organ-specific factors and interactions in determining the successful establishment and propagation of metastatic cancer cells within a particular organ [Figure 3].

In 2015, a study by Hoshino *et al.* was the first to provide support for Paget’s hypothesis, demonstrating that tumor-derived EVPs could be home to future metastatic niches in specific organs^[26]^. For example, EVPs carrying integrin αvβ5 were found to target the liver, while EVPs expressing α6β1 and α6β4 integrins preferentially homed to the lungs^[26]^. Furthermore, EVP education with lung tropic breast cancer derived-EVPs was found to be sufficient to allow otherwise bone metastatic cells to grow in the lung. Additionally, the knockdown of integrin β4 on lung tropic cells and their EVPs was sufficient to reduce the lung metastatic capacity of breast cancer cells. Moreover, EVP integrins, specifically α5, can also mediate breast cancer cell homing to the bone and communication with osteoblasts^[51]^. By promoting osteogenic differentiation of osteoblasts, EVPs containing α5 integrin may create a more hospitable environment for cancer cells to grow and spread.

In salivary adenoid cystic carcinoma (SACC), integrin α2β1 mediates the uptake of CAF-derived EVPs by lung fibroblasts and activates the TGF-β pathway, resulting in the lung fibroblast activation and expression of PMN proteins, such as fibroblast activation protein, α-smooth muscle actin, and periostin^[50]^. Blocking integrin α2β1 with an inhibitor has been shown to attenuate CAF-EVP uptake by lung fibroblasts, inhibit activation of fibroblasts, and suppress lung PMN formation and subsequent lung metastasis in SACC animal models. These findings suggest that integrin α2β1 plays a crucial role in the formation of lung PMNs. In summary, integrins play a central role in EVP trafficking, homing, and organotropism in the context of metastatic cancer. These discoveries provide opportunities for therapeutic targeting in multiple cancers, enabling the development of organ-specific treatments to prevent metastasis.

EVP microRNAs also play a pivotal role in the process of cancer cell organotropism^[79]^. MicroRNAs are small non-coding RNAs that regulate gene expression post-transcriptionally, thereby controlling cellular processes such as proliferation, migration, and differentiation^[80]^. Consequently, the specific miRNA cargo carried by EVPs dictates the organotropism of cancer cells, guiding their migration and invasion to preferred metastatic sites^[81]^. For example, miR-141-3p^[82]^ and miR-940^[83]^ have been implicated in promoting bone metastasis in prostate cancer through their effects on osteoblast and osteoclast activity, as well as bone resorption. Prostate cancer-derived EVPs containing miR-141-3p are taken up by osteoblasts, where they suppress the expression of the target gene DLC1, a Rho GTPase-activating protein^[82]^. This suppression leads to enhanced osteoblast activity, promoting a favorable environment for prostate cancer cell colonization in bone. Similarly, cancer-secreted EVPs containing miR-940 are internalized by osteoblasts, targeting Rho GTPase activating protein 1 (ARHGAP1) and reticulophagy regulator family member 2 (FAM134A)^[83]^. The downregulation of these genes promotes an osteoblastic phenotype in the bone metastatic microenvironment, facilitating prostate cancer cell colonization and bone metastasis. Additionally, miR-181c modulates breast cancer cell organotropism to the brain by targeting PDPK1 in brain endothelial cells, destroying BBB^[81]^. Finally, miR-122 fosters a metabolic symbiosis in the lung and brain by downregulating pyruvate kinase muscle isozyme M2 (PKM2), supporting metastatic tumor cell growth and survival^[84]^. Together, these mechanisms enable cancer cell metastasis to specific organs, where they can colonize and form secondary tumors. Of note, while some studies have suggested that cancer is associated with an overall reduction in microRNAs in cancer cells and tumor microenvironment, recent research has shown that certain microRNAs are packaged at high levels in EVPs derived from tumor cells^[27,52,61,85,86]^.

In conducting experimental studies, particularly those related to metastasis, administration of EVPs warrants thoughtful consideration, especially regarding dosing^[87]^. Established protocols involve injecting 5-10 ug of EVPs intravenously every other day over a span of three weeks^[25,26,50,51,81-84]^. This approach generates a concentration of EVPs in the blood within the physiological range of blood EVP concentrations in tumor-bearing mice with cancer adequate. However, the production and release rates of EVPs can vary considerably depending on the cancer type, its stage, and the general condition of the tumor microenvironment^[48]^. Given the enormous heterogeneity across different cancer types and the dynamic progression of tumors, it is challenging to determine a universally applicable “per day, per cell” rate of EVP production *in vivo*^[48]^. Moreover, the functional impact of EVPs is not merely dictated by their quantity, but also by the molecular cargo they carry, such as proteins like integrins and non-coding RNAs like microRNAs^[48,88]^. Thus, additional research, including comparative studies of EVP production rates across different tumor types and stages, will be vital to adjust EVP dosing in order to faithfully reflect the *in vivo* situation. Furthermore, the translation of these findings into human clinical applications will require careful dose escalation and rigorous safety testing^[87]^.

In summary, while both microRNAs and integrins in tumor-derived EVPs contribute to cancer organotropism, they do so through fundamentally different mechanisms: microRNAs act by modulating gene expression within recipient cells, whereas integrins mediate EVP-cell interactions and organ-specific homing.

In conclusion, research on the role of EVPs in the formation of PMN has greatly advanced our understanding of metastasis organotropism and provided support for Paget’s “seed and soil” hypothesis. By identifying specific proteins, nucleic acids, and pathways involved in this process, new therapeutic strategies may be developed to target and inhibit the formation of PMN, ultimately improving patient outcomes in various types of cancer.

#### Vasculature

The involvement of EVPs in tumor angiogenesis and vascular remodeling was first recognized in the early 2000s. The first studies demonstrating that tumor-derived EVPs could transfer proangiogenic factors to endothelial cells, promoting blood vessel formation and supporting tumor growth at distant sites were published over a decade ago, when our laboratory demonstrated that melanoma-derived EVPs contribute to metastatic invasion by carrying messenger proteins that direct BMDCs towards a pro-metastatic phenotype, promoting proangiogenic events and modifying ECM at pre-metastatic sites^[25]^. Moreover, a subsequent study by Zhou *et al.* showed that EVP miR-105 targets the tight junction protein zonula occludens-1 (ZO-1), enabling the colonization of distant locations by cancer cells^[52]^. EVPs derived from highly metastatic breast cancer cell lines showed significantly higher miR-105 levels, which were associated with increased endothelial permeability and destruction of vascular sprouts at distant sites. These findings underscore the critical role of tumor-derived EVPs in vascular processes, such as angiogenesis, vascular remodeling, and metastasis^[89]^.

#### Coagulation

The link between cancer and coagulation disorders has been recognized since the 19th century^[90,91]^. However, it was not until the 1980s that researchers began to uncover the role of tumor-derived EVPs in promoting thrombosis^[92,93]^. Tumor cell-derived microvesicles carrying tissue factor (TF), a potent initiator of the coagulation cascade, lead to thrombin generation, fibrin deposition, and clot formation^[94-101]^. Recent studies have also revealed that small EVPs can also promote cancer-associated thrombosis by inducing the formation of neutrophil extracellular traps (NETs)^[102]^, which provide structural support to clot formation, promote thrombin activation, and directly activate platelets have been shown to induce platelet aggregation via NETs^[103]^. As such, levels of plasma citrullinated histone 3, characteristic of NET formation, are particularly elevated in cancer patients and correlated with thrombotic events^[103]^. More research is needed to understand how EVPs contribute to clotting in cancer, with the potential to identify new therapeutic interventions by targeting extracellular vesicle-mediated coagulation in cancer patients.

#### Metabolism

The role of EVPs in modulating cancer metabolism began to emerge in the late 2010s^[104]^, as researchers discovered that CAF-derived EVPs could reprogram the metabolic phenotype of tumor cells^[104]^. These EVPs can transfer oncogenic molecules, such as miRNAs and proteins, to stromal and immune cells, modulating their metabolism to support tumor growth and survival^[105-110]^. For example, Cao *et al.* provide insights into the role of breast cancer-derived EVPs and miR-122 in systemic glucose and insulin dysregulation, by demonstrating that breast cancer-derived EVPs impair insulin secretion and glucose homeostasis^[111]^. Additionally, it reveals that miR-122, which is highly secreted by breast cancer cells, plays a key role in suppressing glycolysis and insulin granule exocytosis in pancreatic β-cells^[111]^. The suppression of glycolysis and insulin granule exocytosis in pancreatic β-cells carries substantial consequences for breast cancer patients. Its suppression can lead to an energy imbalance that affects both healthy and cancerous cells^[112,113]^. Furthermore, the impaired release of insulin due to hindered insulin granule exocytosis disrupts glucose uptake regulation by cells^[111,114,115]^. This disruption can result in dysregulated glucose and insulin levels, potentially causing insulin resistance, increased inflammation, and promoting cancer progression^[116-119]^. Additionally, imbalanced glucose and insulin levels may give rise to metabolic complications, such as diabetes, which negatively impact a patient's overall health and well-being^[120-122]^. This can, in turn, influence the effectiveness of cancer treatments and the patient's quality of life^[123-125]^.

Moreover, EVPs have been shown to facilitate the transfer of metabolites between tumor cells, enabling metabolic heterogeneity and promoting treatment resistance. Current research is focused on developing therapies that target the metabolic reprogramming mediated by EVPs to improve cancer treatment outcomes. Interestingly, Wang *et al.* recently reported that tumor-derived EVPs containing fatty acids, particularly palmitic acid, are taken up by Kupffer cells in metastasis-free livers of tumor-bearing mice and cancer patients, inducing tumor necrosis factor α (TNFα) secretion and dysregulating liver metabolism by altering the expression of cytochrome c-encoding genes^[126]^. This metabolic reprogramming of the liver induced fatty liver generation and enhanced the adverse events of chemotherapy, such as myelosuppression and cardiotoxicity. Interestingly, Exo-S and specifically exomeres (and, to a lesser extent, Exo-L) can dysregulate liver function. This study showed that EVPs systemically affect not only future metastatic sites but also non-metastatic organs, particularly in the liver, by altering lipid metabolism, thereby decreasing liver function.

However, there are other metabolic effects of EVPs in cancer that are worth exploring, as well as additional systemic effects of EVPs beyond metabolic changes. In particular, EVPs can impact amino acid metabolism, which is crucial for cancer cell survival and proliferation, as some cancer cells have been reported to release EVPs containing enzymes involved in amino acid metabolism. This may contribute to the reprogramming of recipient cells and promote tumor growth^[104]^. Furthermore, EVPs derived from cancer cells with dysfunctional mitochondria transfer their mitochondrial DNA and proteins to healthy cells and influence mitochondrial function in recipient cells^[127,128]^, leading to altered energy metabolism and contributing to the development and progression of cancer.

#### Immune System

The interplay between cancer and the immune system has been a topic of intense research for the past four decades ^[129]^. In the early 2010s, studies began to highlight the role of tumor-derived EVPs in modulating immune responses^[25,53-55,74]^.

A pivotal study by Pucci *et al.* uncovered the role of lymph node subcapsular sinus (SCS) macrophages in suppressing melanoma progression, by restricting tumor-derived EVP interactions with B cells^[56]^. Melanoma-derived EVPs are capable of stimulating B cells to create a pro-tumoral environment that supports melanoma growth. However, SCS macrophages capture these tumor-derived EVPs, thereby reducing their ability to interact with B cells and preventing the establishment of a pro-tumoral environment^[56]^. Tumor-derived EVPs carry immune-modulatory molecules, such as programmed cell death-ligand 1 (PD-L1), and to suppress antitumor immune responses by inhibiting the function of T cells, natural killer (NK) cells, and dendritic cells (DCs)^[53,55]^. Furthermore, tumor-derived EVPs stimulate Toll-like receptor 3 (TLR3) activation in lung epithelial cells, which in turn initiate an inflammatory cascade and promote PMN formation^[74]^. These findings indicate that host lung epithelial cell TLR3 and recruited neutrophils cooperate in reprogramming immune cells within the PMN, fostering a pro-metastatic inflammatory microenvironment that supports lung colonization of metastasis-initiating cancer cells. Interestingly, a study by Ortiz *et al.* revealed that tumor-derived EVPs selectively downregulate interferon alpha and beta receptor subunit 1 (IFNAR1) in leukocytes, compromising the immune system's ability to counter tumor metastasis by interfering with the protective IFNAR1-cholesterol 25-hydroxylase (CH25H) pathway, essential for preventing tumor-derived EVP uptake and PMN formation^[54]^. The IFNAR1 receptor is significantly downregulated in leukocytes of melanoma patients with metastatic disease compared to healthy donors or patients without metastases, rendering normal cells more vulnerable to tumor-derived EVP uptake, leading to PMN formation. Additionally, Yin *et al.* explored how tumor-derived EVPs promote the dysfunction of tumor-infiltrating dendritic cells, highlighting that lipid accumulation may drive impairment in priming cytotoxic CD8^+^ T cells^[130]^. The authors demonstrated that tumor-derived EVPs, rich in fatty acids, contributed to the observed lipid accumulation and ensuing immune dysfunction in these bone marrow-derived dendritic cells. Notably, peroxisome proliferator-activated receptor α (PPARα)-dependent metabolic shift in dendritic cells from glycolysis to oxidative phosphorylation (OXPHOS), underlining the significance of PPARα-controlled fatty acid metabolism pathways, and PPARα inhibition ameliorated lipid accumulation and improved dendritic cell immune function. Moreover, cancer-derived EVPs can induce immune suppression by inhibiting T cell activation^[55,131,132]^, NK cell-mediated cytotoxicity^[133]^, and promoting the polarization of macrophages towards a tumor-promoting phenotype^[134-137]^. The discovery of these immunosuppressive mechanisms has spurred the development of combination therapies that aim to target tumor-derived EVPs in conjunction with immune checkpoint inhibitors to enhance antitumor immunity^[138]^.

In conclusion, the field of EVPs has come a long way since their initial discovery in the 1980s, when they were thought to be cellular debris or cellular “garbage disposals”. Over the past few decades, numerous groundbreaking studies have instead revealed the critical roles that EVPs play in cancer progression and metastasis. Their involvement in intercellular communication, promotion of angiogenesis, immune response modulation, EMT, and formation of PMNs has transformed our understanding of cancer progression.

As our knowledge of the complex interplay between EVPs and cancer metastasis continues to grow, new therapeutic strategies targeting EVPs and their cargo may emerge, providing novel approaches to combat cancer progression and metastasis. The ongoing research in this field holds great promise for the development of innovative cancer treatments and a deeper understanding of the mechanisms driving metastasis. One key aspect of this research involves the identification and study of biomarkers, which can help track disease progression and response to therapy. In the next section, we will delve into the role of biomarkers in cancer research and how they may further advance our understanding of EVPs in metastasis.

## EVPs AS CANCER BIOMARKERS

In recent years, the potential of using EVPs as biomarkers for various diseases, including cancer, has gained significant attention^[139]^. The use of EVPs as biomarkers for cancer has several advantages over traditional methods^[140]^. Firstly, detecting EVPs in bodily fluids, such as blood^[141]^, urine^[142]^, or saliva^[143,144]^, is non-invasive, unlike tissue biopsies, rendering large-scale screening of the general population more feasible and less costly. Additionally, EVPs are very abundant in these bodily fluids^[25,141]^ and provide a more accurate representation of the tumor microenvironment and systemic changes than single biomarkers^[48]^, such as proteins or miRNAs, or local sampling such as biopsies. As EVPs carry a diverse range of bioactive molecules, they can better reflect the complexity and heterogeneity of cancer^[48,145]^. Lastly, the stability of EVPs in bodily fluids, conferred by their bilayer lipid membrane, offers a significant advantage over other biomarkers, especially nucleic acid ones, which are often rapidly degraded^[3,146]^. This stability allows for more accurate and reliable testing, as well as the potential for monitoring disease progression and response to therapy.

This potential has not gone unnoticed, and some practical applications have already been approved by the United States Food and Drug Administration (FDA). For example, Exosome Diagnostics, now Bio-Techne, has developed a series of diagnostic tests based on the use of exosomes and other EVPs. Their ExoDx Prostate (IntelliScore) is a urine test that utilizes exosomal RNA to detect three biomarkers [ERG (V-ets erythroblastosis virus E26 oncogene homologs), PCA3 (prostate cancer antigen 3), and SPDEF (SAM pointed domain-containing ETS transcription factor)] for the assessment of prostate cancer risk^[142]^. Compared to serum prostate-specific antigen (PSA), which is a gold standard for monitoring prostate cancer but has low sensitivity and specificity in terms of diagnosing prostate cancer, ExoDx Prostate showed better performance in detecting high-grade prostate carcinoma. This test demonstrates the real-world utility of EVPs as biomarkers in current clinical practices.

Recently, Hoshino *et al.* characterized the complete proteomic profile of EVPs from plasma samples of 16 different cancer types and identified EVP protein cargo overrepresented or downregulated in cancer-associated EVPs^[48]^. These proteins could distinguish between cancer and non-cancer or different types of cancer with high sensitivity and specificity, demonstrating the potential use of EVPs as liquid biopsy markers for early cancer detection^[48,147]^. Notably, the study revealed that cancer-derived proteins were not the potential cancer biomarkers in blood-circulating EVPs, but that approximately 50% of the cancer-associated signature proteins were derived from the tumor microenvironment, distant organs and the immune system. These findings highlight these cancer-associated or induced EVP cargo changes as critical components of early cancer detection biomarker signatures.

The study also found that EVP protein packaging is heterogeneous across tumor types and reflects tumor biology. The researchers then identified a combination of EVP proteins that were most likely to distinguish cancer from non-cancer, which were highly enriched in both pancreatic and lung tumor tissues and predictive in identifying cancer. Furthermore, plasma-derived EVPs could be used as a liquid biopsy for cancer detection and to determine tumor type. Additionally, the researchers identified 29 EVP proteins that had the highest predictive value for distinguishing among four different cancer types: melanoma, colorectal, pancreatic, and lung cancer. These findings suggest that plasma-derived EVP proteomes represent tumor-specific signatures capable of distinguishing cancer types, independent of their stage, and can be beneficial in determining tumor type for a diagnosis in patients with cancer of unknown primary tumor origin.

Additional studies have found correlations between specific EVP contents and clinical outcomes. In patients with primary colorectal cancer, high expression of serum HSPC111-containing EVPs positively correlated with liver metastasis^[148]^, while high expression of circulating transforming growth factor β receptor II (TβRII)-containing EVPs was associated with lower survival rates, as well as lower metastasis-free survival rates. High levels of plasma S100 calcium binding protein A4 (S100A4)-containing EVPs and osteopontin in patients with hepatocellular carcinoma are associated with worse survival and disease-free survival^[149]^. Patients with high levels of both S100A4 and osteopontin also have a worse prognosis. Finally, in melanoma patients, high levels of pigment epithelium-derived factor (PEDF)-containing EVPs are associated with higher survival rates^[150]^.

Nucleic acids packaged within EVPs, such as RNA, especially microRNAs, are another class of molecules that can be used as diagnostic and prognostic biomarkers for various types of cancer^[27,52,61,85,86]^, providing valuable information for cancer detection, diagnosis, prognosis, and monitoring. EVP DNA, including double-stranded DNA fragments representing cancer-specific DNA mutations or abnormal copy number variations, can be detected in the blood or other bodily fluids of cancer patients and thus can function as a cancer biomarker^[142,151-154]^. Moreover, the DNA content of extracellular vesicles can reveal specific genetic alterations or mutations related to a particular type of cancer, which can help in determining the cancer type and its origin, especially in cases of cancers of unknown primary origin. One advantage of EVP-DNA is that it may provide genomic information on distant or occult metastatic sites, or sites that cannot be biopsied^[153,155,156]^. Changes in the levels or composition of EVP-DNA can be associated with cancer progression, aggressiveness, or metastasis. For example, higher concentrations of specific EVP-DNA (such as mutant *KRAS* DNA) may be linked to poor prognosis or advanced stages of cancer^[157,158]^. During cancer treatment, the levels of specific EVP-DNA can be used to assess the effectiveness of therapy^[159]^. A decrease in cancer-related EVP-DNA could indicate a positive response to treatment, while an increase or lack of change might suggest resistance or a need to modify the treatment strategy^[160-162]^.

In recent decades, the field of extracellular vesicles has made significant strides, with the potential of EVPs as biomarkers for cancer type, stage, and outcomes becoming increasingly evident. Current research focuses on refining methods for isolating and characterizing EVPs, identifying new and specific biomarkers, and exploring EVP-based therapies, such as cancer-targeting drug delivery or gene silencing. To fully realize the potential of extracellular vesicles in cancer detection, prognosis, and therapeutic guidance over the coming decade, researchers must address several challenges and take crucial steps. Prioritizing studies with larger, multi-center, diverse cohorts will improve biomarker selection and uncover novel biomarkers for various cancer types and patient populations. Developing accessible and standardized methods for EVP isolation and characterization is essential for enabling comparisons between studies and increasing the robustness of findings. Investigating the specificity and sensitivity of biomarkers in EVPs is crucial for accurate and reliable results. By focusing on these challenges, the potential of EVPs as powerful tools for cancer detection, prognosis, and therapeutic guidance can be fully realized, ultimately leading to improved patient outcomes and more effective treatments.

## THE THERAPEUTIC POTENTIAL OF EVPs

The ability of EVPs to act as vehicles for delivering various biomolecules has prompted extensive research into the therapeutic applications of EVPs^[17,163]^. The pioneering study aiming to exploit EVPs for therapeutic purposes used EVPs derived from DCs pulsed with tumor antigen peptides as a vaccine^[164]^. This study demonstrated that EVP-based therapy could prime the function of T cells and exert antitumor effects. Although the subsequent clinical trials did not achieve the predefined efficacy, this landmark study demonstrated the potential for using EVPs in cancer therapy and showed that immune cells and other non-cancerous cells could also be targeted for cancer therapy using EVPs. Subsequently, Alvarez-Erviti *et al.* genetically engineered DCs to express a fusion protein of an EVP membrane protein and a neuron-affinity protein, resulting in the successful production of neuron-targeted EVPs^[165]^. Loading these neuron-affinity-enhanced EVPs with siRNA reduced the expression of Alzheimer's disease target proteins in the brain. The approach of genetically modifying cells to alter the membrane surface of the produced EVPs and loading cargo to exert effects on target cells demonstrates the potential of engineering EVPs to selectively deliver biomolecules to target cells and organs, inducing intended biological effects.

Engineered EVPs carrying proteins, nucleic acids, small molecules, and anticancer drugs can induce various biological effects in target cells. For example, siRNA targeting the *Kras* G12D mutation was introduced into fibroblast-like mesenchymal cell-derived EVPs via electroporation, inhibiting tumor growth in a preclinical pancreatic cancer mouse model and serving as a basis for clinical trials^[166,167]^. EVPs loaded with anticancer agents, such as doxorubicin, also exhibit antitumor effects^[168]^. Beyond merely transporting biomolecules, EVPs can mediate targeted gene editing in cells when loaded with CRISPR-Cas9^[169]^, suggesting an increased potential for EVP-based therapeutic approaches.

Furthermore, EVPs with engineered surface components can selectively target specific cells. For example, rabies viral glycoprotein (RVG) has a high affinity for neurons, and expressing RVG on the surface of genetically modified EVPs targets them to the central nervous system^[165]^. Additionally, enrichment of specific combinations of integrins may enable the development of EVPs that can be taken up by selected target cells. As such, engineering not only the payload but also the surface of EVPs is expected to be increasingly utilized to fully harness their properties.

Highly engineered EVPs, such as enveloped protein nanocages (EPNs), which contain artificial proteins nanocages^[170]^, have also been developed. The production of protein nanocages mimics viral assembly, autonomously assembling within cells and then trafficking through vesicles. EPNs, enveloped by a lipid bilayer, are then released from the cells. EPNs contain tens of protein nanocages, which can deliver biomolecules to target cells and exert their effects. Numerous strategies for engineering EVPs are being developed, and it is expected that they will become mainstream in the future. Combining engineered EVPs with other treatment modalities, such as chemotherapy, radiotherapy or immunotherapy, may also represent promising anticancer strategies.

In addition, EVPs derived from specific cells, such as mesenchymal-derived stem cells (MSCs), dendritic cells, platelets, adipocytes, and even plants, are being studied as sources of EVPs for treatment^[171]^. Particularly, EVPs derived from MSCs may be effective in immune modulation and cell regeneration, and their application in neurological and cardiovascular diseases is being investigated.

Research is also being conducted to evaluate the inhibition of EVP production or uptake to improve pathological conditions propagated by EVPs themselves^[163]^. Cancer-derived EVPs expressing PD-L1 block antitumor immunity by interacting with programmed cell death-1 (PD-1) on immune cells, reducing the efficacy of immune checkpoint inhibitors (ICBs)^[53,55]^. Hence, a combination of a peptide that destroys tumor-derived EVPs expressing PD-L1 and ICB has been shown to improve antitumor immunity in the mouse model^[138]^. This demonstrates the success of strategies that target EVPs themselves for treatment.

In summary, as our understanding of the pathophysiology of EVPs advances, research on applying EVPs for therapeutic purposes is rapidly progressing. Notably, ongoing research aims to enhance the functionality of EVPs, not only by incorporating various biomolecules and compounds as cargoes, but also by modifying the EVPs themselves. Nevertheless, the clinical application of engineered EVPs presents several challenges, including assessing their efficacy and safety in preclinical trials, selecting appropriate source cells and isolation methods, establishing clinical-grade manufacturing, and standardizing dosing and administration routes. Despite the aforementioned challenges, therapies involving EVPs have advanced to the stage of clinical trials, and it is expected that their applications will continue to expand in the future.

## TECHNOLOGICAL ADVANCES ENABLING EVP RESEARCH

The progress in EVP research cannot be discussed without considering technological advancements, including the isolation and purification of EVPs and analysis of single EVPs. In this section, we highlight the technological progress made in EVP analysis over the last decade.

### Isolation and purification of EVPs

A variety of EVP isolation methods exist based on various EVP properties: traditional differential ultracentrifugation and ultracentrifugation using density gradient, for example, rely on size and density differences^[172,173]^. More recently, size exclusion chromatography (SEC) and ultrafiltration have also emerged as size-based separation methods^[174,175]^. Other methods based on surface markers, such as immunoaffinity capture (IAC), and polymer precipitation using commercially available kits, have also been developed. Recently, asymmetric flow field flow fractionation (AF4) emerged as a method to separate small-size EVPs that contain various populations^[12,14]^. AF4 controls two streams flowing through a thin channel with a semi-permissive bottom wall membrane, allowing separation of EVPs ranging from a few nanometers to 100 nm in size. AF4 has enabled the separation of ENPs smaller than 50 nm that were difficult to separate using traditional methods, facilitating the characterization of ENP cargo and function. Microfluidic-based technologies are also gaining attention as a method to separate and analyze EVPs from small sample volumes^[176,177]^. By flowing the sample through microchannels on the micron scale and detecting EVPs based on surface markers or size, it is possible to detect and analyze EVPs from limited samples at high throughput, rendering this method highly sought-after. Each separation method has its own advantages and disadvantages, and a standard method has not been established^[16,178]^. The main challenges are how to effectively separate heterogeneous EVPs, prevent co-isolation of non-EVP material (such as aggregate proteins, viruses, and lipoproteins), prevent the loss of EVPs during separation, and minimize time and cost. Furthermore, the optimal separation method should be chosen according to the sample source. For instance, plasma samples contain a variety of impurities and require more advanced separation methods. For biomarker discovery and detection, it is desirable to use methods that can separate EVPs from small amounts of samples, while for therapeutic purposes, it is necessary to consider methods that can efficiently separate large amounts of EVPs.

### Single EVP analysis

The growing awareness of EVP heterogeneity has revealed limitations in conventional bulk analysis approaches. As a result, there is an increasing need for analyses focusing on EVP subpopulations and even single EVPs, which have become more common over the past decade^[179]^. Nanoparticle tracking analysis (NTA) is a method for analyzing the size of isolated EVPs by imaging the scattered light caused by the particles' Brownian motion^[180]^. This allows for label-free determination of EVP size (ranging from 70 nm to several hundred micrometers) and particle count in solution. Additionally, high-resolution flow cytometry is gaining popularity^[181]^. By staining surface antigens with fluorescently labeled antibodies, single EVP characterization is possible. Furthermore, a proximity barcoding assay was recently used to analyze the expression of individual molecules for single EVPs^[182]^. This was accomplished by conjugating a DNA barcode to an antibody that recognizes molecules expressed on the surface of EVPs, allowing for high-resolution expression analysis.

Imaging techniques for single EVPs include not only conventional transmission electron microscopy but also atomic force microscopy (AFM) for analyzing EVP surface structures^[183]^. AFM allows for observation at high resolutions, such as a vertical resolution of approximately 0.1 nm. Furthermore, super-resolution fluorescence microscopy [photoactivated localization microscopy (PALM) and stochastic optical reconstruction microscopy (STORM)] has become available in recent years and is playing an increasingly significant role in single EVP analysis^[184]^. This versatile technique can be used for various applications, such as staining and observing surface antigens at the single EVP level and tracking EVP trafficking within cells. As a means of analyzing the composition of single EVPs, research using laser tweezers Raman spectroscopy (LTRS) has also been reported^[185,186]^. LTRS is a nondestructive method for analyzing molecular structures based on Raman scattered light, and it can examine the composition of proteins, nucleic acids, and lipids in single EVPs. By combining LTRS with other techniques, it has been demonstrated that tumor-derived EVPs can be distinguished from erythrocyte and platelet-derived EVPs in plasma at the single EVP level^[187]^. Moreover, combining EVP labeling and microscopy has enabled *in vivo* single EVP analysis^[188-191]^.

To summarize, various analytical methods for single EVPs have become available over the past decade, revealing characteristics of EVPs that were previously unknown due to the limitations of bulk analysis. Additionally, observing and tracking the intracellular localization of specific EVPs have become useful for exploring biogenesis. *In vivo* single EVP analysis has also become possible^[190]^. The emergence of these technologies has deepened our understanding of EVP heterogeneity. However, many of these technologies are not suitable for high-throughput single EVP analysis and the development of such methods is much needed. While proteomics and genomics analyses have been applied to bulk EVPs, single EVP “omics” analysis remains challenging, and the development of systems capable of omics analysis with minimal input is desirable. Thus, analysis techniques targeting single EVP will continue to advance and serve as a driving force for future EVP research.

## DISCUSSION

Over the last decade, the EVP-cancer field has experienced significant advancements that have fundamentally changed our understanding of intercellular communication and cancer biology. With the convergence of state-of-the-art technologies, interdisciplinary collaboration, and novel insights, the field of EVPs will continue to undergo a paradigm shift in the next decade, unlocking new possibilities in cancer research and treatment. Looking back, the discovery of EVPs as key players in cell-to-cell communication has paved the way for a deeper understanding of their roles in various physiological processes and diseases, particularly cancer progression and metastasis. Key processes involving EVPs include metastasis, angiogenesis, immune response modulation, epithelial-to-mesenchymal transition, and PMN formation. Furthermore, the identification of various EVP-associated biomolecules, including proteins, lipids, and nucleic acids, has shed light on their diverse functions and potential as therapeutic targets.

We can expect groundbreaking discoveries that will redefine our understanding of EVPs and their roles in cancer biology for decades to come. The discovery of novel EVP subtypes with unique properties and functions will revolutionize diagnostics, prognostics, and therapeutics. Researchers may uncover EVPs capable of transporting not just biomolecules, but also energy or information through unconventional means, such as quantum communication or other yet-to-be-discovered phenomena. The integration of cutting-edge technologies, such as nanotechnology, synthetic biology, and artificial intelligence, will play a pivotal role in shaping the future of EVP research. Nanotechnology will enable the creation of hybrid, bioengineered EVPs with the ability to self-assemble, replicate, or even harness energy from their environment. These smart, programmable EVPs will have unparalleled therapeutic potential, precisely navigating complex biological systems, responding to environmental cues, and adapting to the ever-changing human body. Synthetic biology will empower researchers to engineer customized EVPs for tailored therapeutics, paving the way for personalized medicine in cancer treatment. By leveraging the immunomodulatory properties of EVPs, advances in immunotherapy will lead to more effective cancer treatments, targeting specific signaling pathways, cellular processes, and even the biophysical properties of the extracellular matrix. Artificial intelligence and machine learning systems will revolutionize EVP analysis and manipulation, allowing researchers to predict and optimize their behavior in real time. These systems will detect subtle patterns and relationships within vast datasets, leading to the identification of novel biomarkers and therapeutic targets previously invisible to researchers. The interdisciplinary collaboration between researchers in the fields of physics, chemistry, materials science, and engineering will further fuel innovation in the EVP-cancer field. For instance, the development of new materials and surfaces for efficient EVP isolation and characterization will improve the reproducibility and accuracy of EVP research. Moreover, advanced imaging techniques, such as super-resolution microscopy and real-time *in vivo* imaging, will provide invaluable insights into the molecular mechanisms underlying EVP biogenesis, cargo packaging, and cellular uptake.

In conclusion, the EVP-cancer field has made remarkable strides in our understanding of intercellular communication and cancer biology. The future of EVP research promises to revolutionize diagnostics, prognostics, and therapeutics, with the potential to transform cancer treatment and patient outcomes. As we continue to unravel the complex roles of EVPs in health and disease, we will undoubtedly uncover novel approaches and applications, reshaping our understanding of the intricate interplay between cells, tissues, and organs, and, ultimately, our ability to combat cancer and other devastating diseases.

## DECLARATIONS

### Copyright

© The Author(s) 2023.
